# Visual signal evolution along complementary color axes in four bird lineages

**DOI:** 10.1242/bio.052316

**Published:** 2020-09-18

**Authors:** Anand Krishnan, Avehi Singh, Krishnapriya Tamma

**Affiliations:** 1 Department of Biology, Indian Institute of Science Education and Research, Pashan Road, Pune 411008, India; 2 Reed College, Portland, OR 97202, USA; 3 Centre for Ecological Sciences, Indian Institute of Science, Bengaluru 560012, India

**Keywords:** Birds, Plumage evolution, Visual signals, Color patterns, Complementary colors

## Abstract

Avian color patterns function in varied behavioral contexts, most being produced by only a handful of mechanisms including feather nanostructures and pigments. Within a clade, colors may not occupy the entire available space, and incorporating complementary colors may increase the contrast and efficacy of visual signals. Here, we describe plumage patterns in four ecologically and phylogenetically diverse bird families to test whether they possess complementary colors. We present evidence that plumage colors in each clade cluster along a line in tetrachromatic color space. Additionally, we present evidence that in three of these clades, this line contains colors on opposite sides of a line passing through the achromatic point (putatively complementary colors, presenting higher chromatic contrast). Finally, interspecific color variation over at least some regions of the body is not constrained by phylogenetic relatedness. By describing plumage patterns in four diverse lineages, we add to the growing body of literature suggesting that the diversity of bird visual signals is constrained. Further, we tentatively hypothesize that in at least some clades possessing bright colors, species-specific plumage patterns may evolve by swapping the distributions of a complementary color pair. Further research on other bird clades may help confirm whether these patterns are general across bird families.

## Abstract (precis)

**Summary:** In four diverse bird radiations, we reveal the presence of complementary colors, putatively for higher contrast. Color patterns may have diversified by redistributing a complementary color pair over the body.

## INTRODUCTION

Avian color patterns represent important visual signals that serve diverse functions (Alatalo et al., 1994; Baker and Parker, 1979; Bleiweiss, 2004; Greene et al., 2000; Seddon et al., 2013; Uy et al., 2009), and their diversification remains an important area of research (Doutrelant et al., 2016; Mason et al., 2014; Stoddard and Prum, 2008). Most bird colors result from a relatively small number of pathways, primarily dietary carotenoids which contribute mainly bright, long-wavelength colors (Hill et al., 2002; McGraw and Nogare, 2004), feather nanostructures that contribute short-wavelength colors (Hill and McGraw, 2006; Saranathan et al., 2012), and melanins that contribute brown, gray and black colors (Hill and McGraw, 2006). Multiple studies have shown that plumage (and egg) colors in birds do not occupy the entire available color space, as defined using tetrachromatic color space models (Cassey et al., 2008; Endler et al., 2005; Stoddard and Prum, 2011), putatively because the nature of color-producing mechanisms limits the number of ways by which a bird may develop contrasting signals. This poses important consequences for the diversification of visual signals. In addition to these, the visual systems and opsin tuning of birds and their predators (Gomez and Théry, 2007; Gotmark, 1993; Marchetti, 1993), and the composition of ambient light in different habitats (Boughman, 2002; Endler, 1992) may influence the contrasts provided by plumage color patterns (Endler, 1992; Gomez and Théry, 2007). How, within the limits imposed by a few major color-producing pathways, do birds evolve contrasting visual signals (Hill and McGraw, 2006)?

Many studies aimed at quantifying color use color space models, which typically involve mapping measured colors onto axes centered around an ‘achromatic point’, with more saturated (or vivid) colors being further away from this point (Wilkins and Osorio, 2019). Colors that are far apart in chromatic space (e.g. on opposite sides of the achromatic point) have little spectral overlap. Thus, different patches exhibiting these colors may excite distinct sets of photoreceptors (Endler, 1992; Endler et al., 2005; Ham and Osorio, 2007). In color space, the extremes of this continuum on either side of the achromatic point represent complementary colors, offering high contrast and discriminability when combined together, particularly over adjacent body regions (Endler, 1992; Endler and Mielke, 2005; Hill and McGraw, 2006; Osorio et al., 1999a). For example, in forest canopy birds, dwelling against a primarily green background, blue colors serve to increase the contrast of red colors against the background (Endler, 1992). In addition to chromatic signals, luminance signals (achromatic or black-and-white variance) are also important to consider as they offer high contrast (Griggio et al., 2011; Marchetti, 1993; Mennill et al., 2003).

Relatively few bird clades have had their overall color space quantified across species, and much remains to be understood about patterns of color space occupancy. It is thus important to describe the color space of additional bird clades to examine which regions of tetrachromatic color space they occupy (Endler and Mielke, 2005; Goldsmith, 1990). This further enables us to examine the distributions of colors for each species within a clade, to test the hypothesis that contrasting signals represent complementary colors. When this analysis is carried out, we predict colors on opposite sides of the achromatic point will represent complementary colors (Endler, 1992; Ham and Osorio, 2007). Colors lying on opposite sides of the achromatic point (i.e. complementary colors) may be expected to exhibit opposite signs from each other if we score them such that the achromatic point lies at zero. Thus, the distributions of maximum and minimum scores for all species within a clade should lie on opposite sides of this point. We thus predict that each species will possess colors on both sides of the achromatic point to increase contrast. Finally, it is important to test whether the presence of complementary colors indicates phylogenetic constraints due to shared ancestry. Closely related species may resemble each other in terms of plumage color placement as a result of this shared ancestry, so phylogenetic comparative analyses will help us ascertain how much color patterns have changed across species. We predict that if complementary colors have supported pattern diversification, then color scores across a clade should exhibit low phylogenetic signal (that is, species resemble each other less than expected by chance). This would rule out the possibility that all species in a clade possess similar patterns, and instead indicate that patterns have diversified by redistributing a complementary color pair across body regions.

**Fig. 1.**
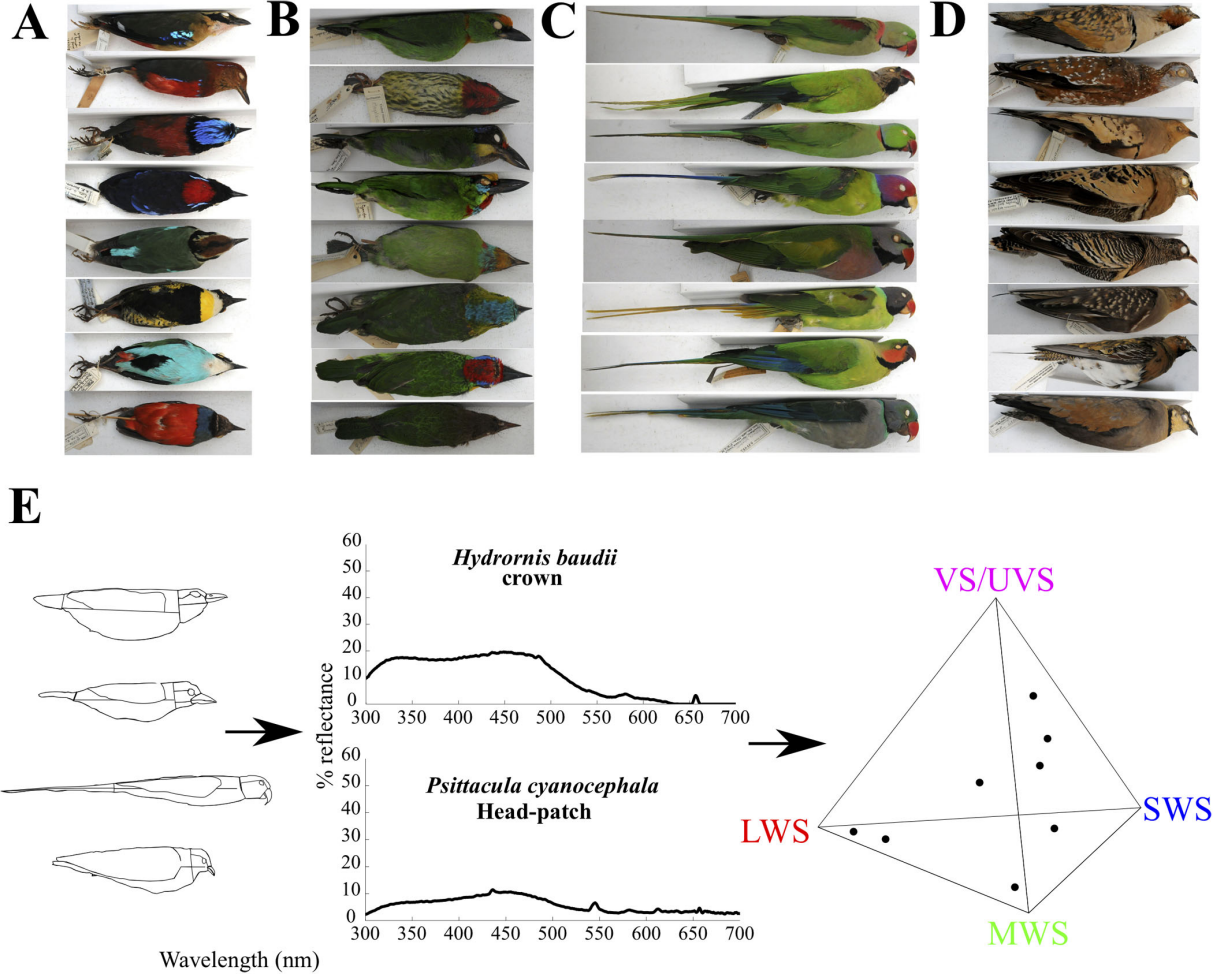
**(A–D) Representative museum specimens of the four bird clades examined in this study, the pittas (A), barbets (B), parakeets (C) and sandgrouse (D), from the collections of the Division of Birds, Smithsonian National Museum of Natural History, Washington D.C., USA.** (E) Workflow of analyses. Using museum specimens from all four clades (left; the regions of the body are demarcated by lines), we measured reflectance spectra (examples in center), and analyzed them using theoretical models of avian color vision including Goldsmith's tetrahedral color space (right), where each vertex represents maximal relative excitation of one of the four cones (and therefore saturated colors).

Here, we describe the interspecific color space in four ecologically and phylogenetically diverse bird clades, using ultraviolet (UV)-visible light reflectance spectrometry (Hill and McGraw, 2006). They are: (1) Pittas (Pittidae), understory invertebrate-eaters occurring from Africa to Australasia (Erritzoe and Erritzoe, 1998); (2) Asian barbets (Megalaimidae), tropical forest-canopy frugivores (Short and Horne, 2001); (3) Afro-Asiatic *Psittacula* parakeets (Psittacidae), fruit and seed-eaters inhabiting deciduous forests and woodland (Forshaw and Cooper, 1989), and (4) Sandgrouse (Pteroclidae), arid-country ground-dwelling granivores (Maclean, 1996) (Fig. 1A–E). These families putatively represent both ultraviolet-sensitive (UVS; parakeets and sandgrouse)- and violet-sensitive (VS; barbets and pittas)-type avian visual systems (based on published information, although choice of model did not influence patterns we observe, see Fig. S1) (Ödeen and Håstad, 2013; Stoddard and Prum, 2011). We first describe the color space occupied by each clade. We note here that our comparisons are within-clade only for four representative groups, and are not a broad comparison across clades. Secondly, we examine within-species patterns in color or luminance scores across body regions and species, and test our hypothesis that they exhibit complementary colors (i.e. lying on opposite sides of the achromatic point), and quantify phylogenetic signal in color scores to test whether the presence of these colors indicates a diversification of color patterns by redistributing complementary colors on the body. By examining the color space in clades occupying different habitats and light environments, we seek to test broadly for the presence of complementary colors.

## RESULTS

### Plumage colors occupy distinct regions in tetrachromatic color space

**Fig. 2.**
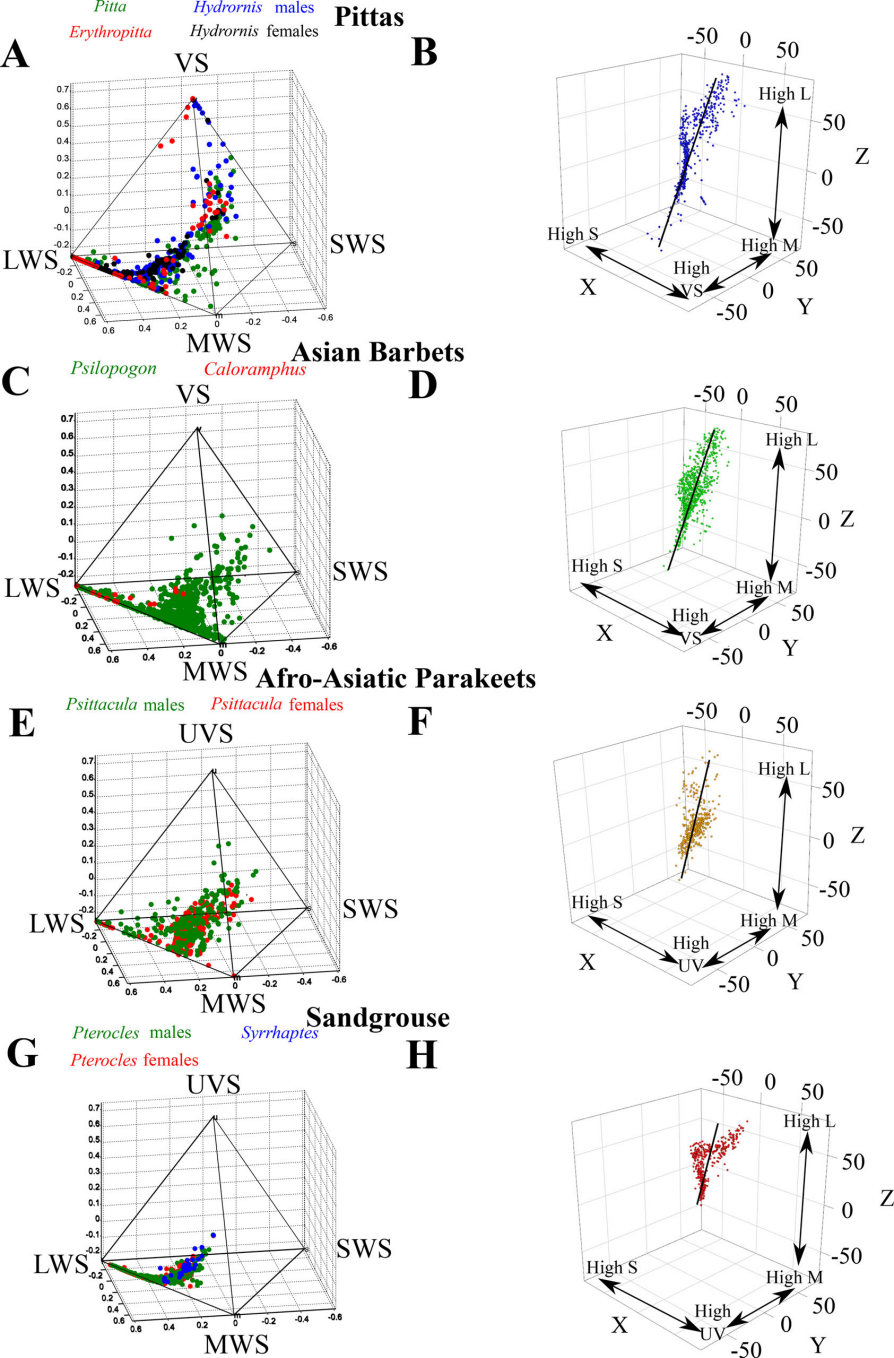
**Color space occupancy and analyses of signal variation in pittas (A,B), barbets (C,D), parakeets (E,F) and sandgrouse (G,H).** Left-hand side figures represent color space occupied by each family (one point/color patch measured), as visualized using Goldsmith's tetrahedral color space. Each vertex represents relative photon catch of a particular cone (see Fig. 1). Right hand side plots represent the same data points transformed into a three-dimensional XYZ color space using a noise-limited model of avian tetrachromatic vision. The black lines through the points represent the first major axis (PC1) of chromatic variation. The different genera and sexes are color coded in the left hand figures for the sake of comparison, and do not correspond to the colors on the right hand plots.

Across the four avian clades, we find that plumage colors distribute between two points in tetrahedral color space. The color signals of pittas lie between red (LWS) and violet (VS) color vertices (indicating highly-saturated colors) (Fig. 2A) (*Hydrornis* occupies 36.03% of the volume of total avian color space, *Erythropitta* 29.92%, and *Pitta* 26.93%). Barbets largely distribute between the green (MWS) and red (LWS) vertices, with a few blue-violet patches (Fig. 2C) (the basal *Caloramphus*: 0.51% of total avian color space, *Psilopogon*: 19.28% of color space). Plumage colors of *Psittacula* parakeets lie between the middle of red-green space and the middle of blue-UV space (Fig. 2E), with a few patches near the LWS and MWS vertices (males occupy 24.03% of avian color space, females 15.86% in this sexually dimorphic clade). Finally, the plumage colors of sandgrouse are restricted to a region between the black achromatic point (the centroid) (Stoddard and Prum, 2008) and the LWS vertex (Fig. 2G) (*Syrrhaptes*: 0.35% of avian color space, *Pterocles* males: 2.96%, *Pterocles* females: 2.19%). An XYZ color space using a noise-limited model of color space (see the Materials and Methods) recovers a linear axis that explains the bulk of color variation, suggesting that plumage colors are largely distributed along an ‘axis’ in tetrachromatic color space. The results of principal components analysis to quantify the proportion of variation explained by this line are summarized below for each avian clade, and also in Table S1 (see Supplementary Data).

#### Pittas

PC1 (the major axis of variation) of the XYZ coordinates in color space explains 85% of chromatic variation (Fig. 2B). PC1 loads weakly negatively on X (−0.15), and exhibits strong positive loadings (0.6 and 0.78) on Y and Z, respectively.

#### Barbets

PC1 explains almost 74% of chromatic variation (Fig. 2D), loading weakly negatively on X (−0.03), moderately positively on Y (0.465), and strongly positively on Z (0.884).

#### Parakeets

PC1 explains 75% of variation in color (Fig. 2F), loading weakly negatively on X (−0.2), moderately positively on Y (0.57) and strongly positively on Z (0.8).

#### Sandgrouse

PC1 explains about 70% of color variation, loading weakly negatively on X (−0.32) and strongly positively on Y and Z (0.64 and 0.7).

Across all four clades, the Z coordinate loads most strongly on PC1, thus suggesting that most variation in perceptual coordinate space occurs along the elevational rather than azimuthal direction along the PC1 line. Therefore, in subsequent analyses, we transformed this XYZ space into a spherical coordinate system, and used the elevational coordinate Φ and the sign of this coordinate as an indicator of where different colors lie along this line. Although this does not take variation in the azimuthal plane into account, the results of our analysis suggest that this variation is relatively low compared to variation along the elevational axis in all four families. Thus, colors with opposite signs of Φ in this dataset should lie on opposite sides of the achromatic point (as is evident from the spread of the data in Fig. 2).

### Colors along the axis lie on opposite sides of the achromatic point

Using species averages for color and luminance scores across body regions, we tested whether (1) species within each clade exhibit colors lying on both sides of the achromatic point, and (2) whether the distribution of colors along a major axis is constrained by phylogenetic relationships (i.e. a lack of phylogenetic signal across at least some body regions). In summary, we uncover evidence that most species in three of the four avian clades (with the exception of the sandgrouse) possess color scores lying on either side of the achromatic point, indicating complementary colors within each species, putatively for higher contrast. Further, phylogenetic comparative analyses suggest that phylogeny does not appear to explain significant variation in color and luminance scores. Regions with high color variation exhibited low or non-significant phylogenetic signal throughout. We summarize the results of these analyses below.

#### Pittas

**Fig. 3.**
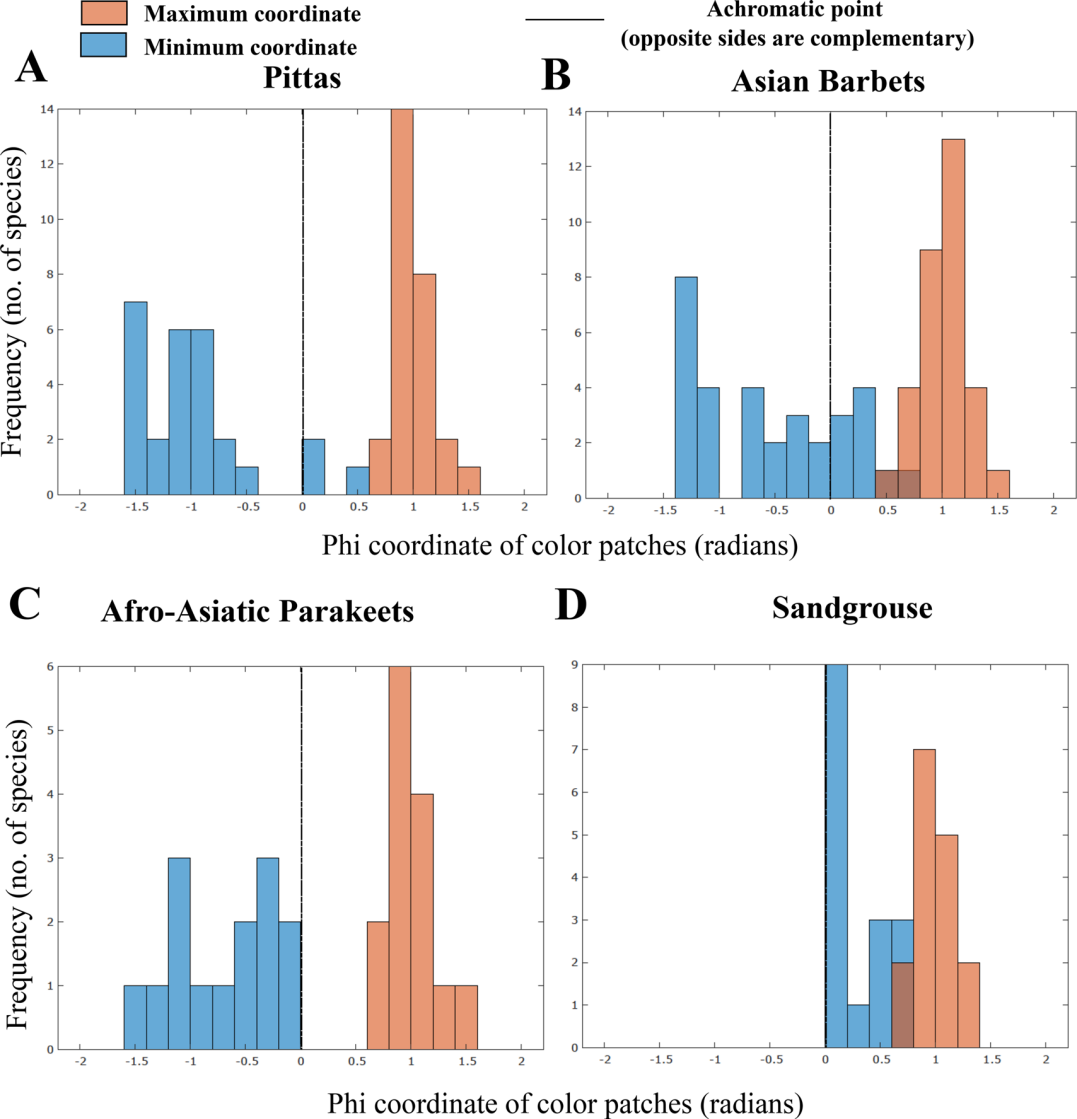
**Linear axes of plumage variation represent complementary colors.** Shown here are histogram distributions of maximum (red) and minimum (blue) color scores (the maximum and minimum phi-coordinate in radians) of each species within four bird clades, the Pittas (A), Asian barbets (B), Afro-Asiatic parakeets (C) and Sandgrouse (D). In the first three families, most species possess colors that are on opposite sides of the achromatic point (0). In the fourth, the sandgrouse, colors are clustered on one side of the achromatic point.

After transforming into a spherical coordinate space, elevation coordinates span between −1.54 and +1.57 across the family, i.e. on opposite sides of the achromatic point and at roughly equal distances from it along the elevation axis. For example, the deep-blue (to human eyes) crown of the male *Hydrornis baudii* has, on average, a color score of −1.15, and the deep-red crown of the sympatric (Erritzoe and Erritzoe, 1998) *Erythropitta granatina* scores +1.12. These colors also represent opposite ends of the avian-visible light spectrum, and thus are highly contrasting with little spectral overlap. Histograms of maximum and minimum color scores of each species within the family show that most of these species possess colors lying on opposite sides of the achromatic point (Fig. 3A) (dF=26, one sample *t*-tests, maximum: t=29.67, *P*<0.001, minimum: t=9.79, *P*<0.001, paired *t*-test: t=19.9, *P*<0.001). Color scores are consistent with a Brownian motion model of evolution on the cheek, wing and tail, and exhibit weak and non-significant phylogenetic signal across other body regions (Table 1). Luminance scores exhibit significant phylogenetic signal only on the crown and wing, and no evidence of phylogenetic constraints on other body regions (Table 2). Mantel tests for correlation between phylogenetic and trait distance broadly corroborate these results: luminance distance correlates significantly with phylogenetic distance only on the wing, whereas color correlates on the cheek, wing and tail (Supplementary Data). In addition, the regions with non-significant phylogenetic signal all possess relatively high coefficients of variation in color scores (Table 1). Thus, patterns of plumage evolution are heterogeneous across the body regions of pittas, with a lack of phylogenetic signal on several body regions.

**Table 1.**
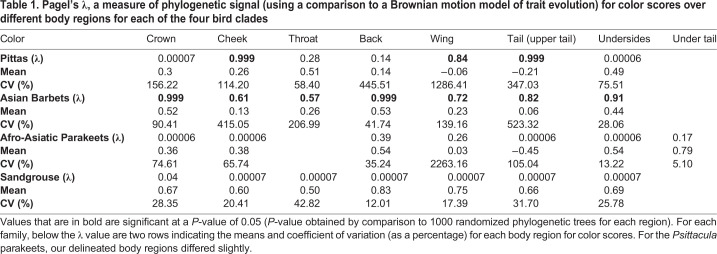
**Pagel's λ, a measure of phylogenetic signal (using a comparison to a Brownian motion model of trait evolution) for color scores over different body regions for each of the four bird clades**

**Table 2.**
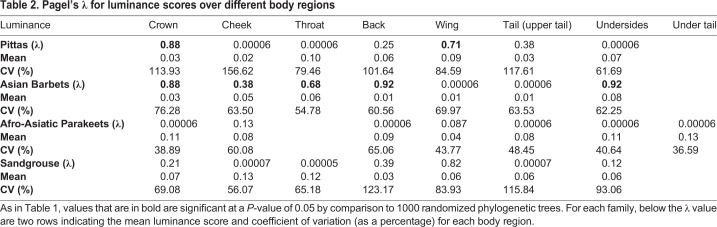
**Pagel's λ for luminance scores over different body regions**

#### Barbets

Color scores span between −1.4 and +1.57, and thus both sides of the achromatic point. For example, the red throat of *Psilopogon mystacophanos*, exhibits, on average, a color score of +1.1, and the turquoise throat of the sympatric (Short and Horne, 2001) *P. rafflesii* a score of −1.21, which, like pitta colors, lie on opposite sides of the achromatic point. Again, histograms of color distribution (Fig. 3B) demonstrate that most barbet species exhibit colors lying on opposite sides of the achromatic point (dF=31, one sample *t*-tests, maximum: t=28.24, *P*<0.001, minimum: t=4.9, *P*<0.001, paired *t*-test: t=13.63, *P*<0.001). Color and luminance scores (Table 1) exhibit significant λ (phylogenetic signal) values across all regions (except luminance scores on the wing and tail), but values for head patches (particularly the cheek and the throat) are much lower than 1 (0.61 and 0.57), indicating overdispersion compared to a Brownian motion model of trait evolution. Phylogenetic and color distance are correlated on all body regions, but not on the head regions (Supplementary Data), corroborating the results from phylogenetic signal. In addition, all head regions possess relatively high coefficients of variation (CVs) for color scores, but not body regions (except the wing, which does, however, exhibit phylogenetic signal suggesting that this variation has a phylogenetic component). Taken together, these results are also consistent with body colors being a constrained feature within this clade, but colors diversifying on the head regions.

#### Parakeets

Color scores span between −1.47 and +1.57. For example, the wing of the male *Psittacula longicauda nicobarica* (−1.38) exhibits the opposite sign to the red shoulder patch of male *P. cyanocephala* (+1.32). Color histograms again indicate that all species possess colors lying on opposite sides of the achromatic point (Fig. 3C) (dF=13, one sample *t*-tests, maximum: t=16.46, *P*<0.001, minimum: t=5.62, *P*<0.001, paired *t*-test: t=12.91, *P*<0.001). Neither color nor luminance scores exhibit significant phylogenetic signal across any body regions (Table 1) when compared to a Brownian motion model of trait evolution, and additionally do not exhibit significant correlations with phylogenetic distance (Supplementary Data). Color scores exhibit higher CVs than luminance scores (Table 1).

#### Sandgrouse

Color scores span between 0 (the achromatic point) and +1.33. Sandgrouse are clustered in chromatic space to one side of the achromatic point, further supported by color histograms (Fig. 3D), although both maximum and minimum scores are, across the family, still significantly different from zero (dF=15, one sample *t*-tests, maximum: t=27.72, *P*<0.001, minimum: t=3.47, *P*<0.01, paired *t*-test: t=9.17, *P*<0.001). However, aside from luminance scores on the wing (Table 1), neither color nor luminance scores exhibit significant phylogenetic signal on any of the body regions. In addition, other than color scores on the crown (Supplementary Data), interspecific color and luminance distances are not significantly correlated with phylogenetic distance. Coefficients of variation of color scores (Table 1) are generally lower than those for luminance across body regions, unlike the other three clades. Thus, in sandgrouse, interspecific luminance (black-white) variation may putatively play a greater role in signal diversification. The sympatric *Pterocles alchata* and *P. orientalis* (Benítez-López et al., 2014) represent a noteworthy example of such divergence*.* The black belly of the male *P. orientalis* exhibits an average luminance score of <0.0001, whereas the white belly of the male *P. alchata* exhibits an average luminance score of 0.34.

## DISCUSSION

Across diverse bird families, we thus uncover consistent evidence that plumage colors within a clade do not occupy the entire available color space, in keeping with previous studies. In three clades, each species possesses complementary colors occurring on opposite sides of the achromatic point, and phylogenetic comparative analyses indicate generally low or non-significant phylogenetic signal (except Asian barbets, where only the head regions diverge from a Brownian motion model of trait evolution). Taken together with the distribution of color scores along a single line in color PC space, we suggest that colors on opposite sides of the achromatic point represent complementary colors. Their presence across most species within at least three clades, together with the lack of phylogenetic signal, suggests tentatively that evolutionary diversification of color patterns in at least some bird clades occurs by redistributing a complementary color pair across body regions. We discuss this further below.

### Visual signals and complementary colors

To summarize, we find that plumage colors in each of the four bird clades distribute along an axis between two colors (or regions of the avian-visible spectrum) that are complementary (spanning either side of the achromatic point), except the arid-country sandgrouse whose colors are found to only one side of the achromatic point (we discuss this further below). Additionally, our phylogenetic comparative analyses uncover heterogeneous phylogenetic signal across body regions, suggesting that colors on at least some regions of the body are not phylogenetically constrained. This suggests that the restricted color space occupied by each clade is not the result of related species closely resembling each other. Our study includes only four clades, and not a broader sample of other bird groups. Further studies are required to test these hypotheses across a broader selection of avian clades. However, we do identify our analyses of four diverse, representative clades as a launching point for further broad studies. Although not directly confirmed in our study systems, it is important to note here that tetrachromatic visual systems (avian and reptilian) possess a number of opponent color processes to compare cone outputs (Goldsmith and Butler, 2005; Osorio et al., 1999b; Rocha et al., 2008; Smith et al., 2002; Ventura et al., 2001; Yazulla and Granda, 1973). In human trichromatic visual systems, red-green, yellow-blue and luminance (black-white) opponent comparisons result in all perceived hues occupying a continuum between these perceptually distinct opponent colors (Hurvich and Jameson, 1957). Different opponent mechanisms (or color axes) dominate at various wavelengths and intensities of ambient light, accordingly shifting the perceived color space (the Bezold–Brücke phenomenon) (Boynton and Gordon, 1965). This may represent a putative mechanism enabling discrimination of complementary colors across light environments, although we do not possess the evidence at present (i.e. physiological data) to explicitly test this. Indeed, because birds possess color constancy (Olsson et al., 2016), the effects of such shifts may not be biologically significant.

However, the distribution of colors within three clades (Fig. 3) is broadly consistent with complementary colors in their plumage, putatively for high chromatic contrast and low spectral overlap (Endler, 1992). For at least two clades, pittas and barbets, these colors appear to be redistributed across body regions in different species. In the case of pittas, these regions appear to be the crown, throat, back and underparts. For example, *Hydrornis baudii* possesses a blue crown and underparts, and a reddish-brown back, whereas the sympatric *Erythropitta granatina* possesses a deep blue-violet back and a bright red crown patch and belly. For Asian barbets, this redistribution of colors appears to occur primarily on the head, and an examination of their color patterns supports this. Most members of the family possess largely green bodies, and bright colors are confined to the head regions. It is noteworthy here that relatively few species in these clades are sexually dimorphic (although we measured males and females wherever possible). *Psittacula* parakeets and sandgrouse are sexually dimorphic, but this has little broad effect on the plumage patterns we observed (Fig. 2). Thus, it is likely that interspecific rather than sexual variation in plumage is responsible for the patterns we detect here. However, future studies on larger bird clades should take sexual dimorphism and the effects of sexual selection into account as well.

### Color mechanisms and complementary colors

Comparing the major color-producing mechanisms in birds might help explain some of the patterns observed in our study. For example, the red colors of pittas and barbets are due to carotenoids (Thomas et al., 2014), likely derived from dietary sources (Hill et al., 2002), in contrast to structural short-wavelength colors derived from feather nanostructures (Saranathan et al., 2012). Parakeet pigment colors are due to psittacofulvins (McGraw and Nogare, 2004). Finally, sandgrouse do not possess plumage carotenoids (Thomas et al., 2014), and pigmentation is thus likely to be primarily melanin-based (brown-black). This may constrain plumage diversification to an achromatic complementary axis (or to changes in barring and speckling, which our study did not investigate); albeit with the caveat that luminance variation is difficult to compare using museum specimens. However, a comparison of plumage patterns in sandgrouse (Fig. 1) reveals that many species possess conspicuous black and white patches, whose distributions differ between species. Some possess these patches on the face, others on the wings and belly. Similar patterns of evolution along an achromatic axis may have also likely occurred in other melanin-pigmented bird groups, such as larks, bustards, and coursers, as well as many raptors (del Hoyo et al., 2014), and merit further investigation.

Ecological pressures of sensory drive (for example, crypsis from predators and conspicuousness to intended receivers) may additionally constrain plumage diversity or the position of complementary colors on the body. All four families studied here experience predation, and possess both cryptic colors, and colors that offer maximal contrast in their preferred habitats. For example, blue-violet and saturated reds are very conspicuous against a forest understory background (Siddiqi et al., 2004), and also reds against the green forest canopy, where blue serves to increase within-pattern contrast (Endler, 1992; Gomez and Théry, 2007); these are the colors exhibited by pittas and barbets, which typically occupy these habitats (Erritzoe and Erritzoe, 1998; Short and Horne, 2001) (Fig. 2). Cryptic colors, defined as matching the background in a habitat (Endler, 1992; Gomez and Théry, 2007) (green in tree-dwelling barbets and parakeets, reddish-brown in ground-dwelling pittas and sandgrouse), occur across all four clades in our study, which are additionally noted in the literature as being unobtrusive, camouflaged or difficult to locate within their habitats (Erritzoe and Erritzoe, 1998; Forshaw and Cooper, 1989; Maclean, 1996; Short and Horne, 2001). Microhabitat variation in light composition may influence which colors are the most conspicuous (Endler and Thery, 1996; Uy and Endler, 2004), as well as whether birds use chromatic or achromatic contrasts in pattern discrimination (Endler and Thery, 1996; Schaefer et al., 2006). However, we have not directly measured the light environments inhabited by these species, and this discussion must, therefore, be considered preliminary, pending further studies on these habitats. Additionally, our use of theoretical models assumes that all species within a clade perceive color the same way, whereas some differences between species are likely to exist. Field data are therefore needed to further understand both the predation these birds experience, and the light microhabitats they use.

Color vision is challenging to study comparatively in speciose bird lineages containing rare or range-restricted species, and many of the species we examine are poorly known. Thus, although our study is primarily descriptive of color patterns in four diverse bird clades, and our design does not permit us to conclusively identify the ecological driver of these patterns, we do find that at least three clades possess apparently complementary colors. Based on this, we tentatively hypothesize that color patterns in some bird clades may have diversified by redistribution or replacement of these complementary colors between species. Further research will focus on testing this hypothesis. Additionally, our study did not quantify evolutionary rates or shifts in color scores. With recent analytical frameworks (Clavel et al., 2015), this presents an exciting avenue of research. A broad comparison of complementary colors and their distributions across bird species would provide a suitable system to address the relationship between ecology and plumage diversification.

## MATERIALS AND METHODS

### Museum specimens

We measured museum specimens of four avian clades, held in the collections of the Division of Birds, Smithsonian National Museum of Natural History (USNM), Washington, D.C., USA (total 273 specimens, Supplementary Data). For the pittas, we measured 80 specimens of 27 species [out of 34 according to the previous taxonomy; more recent taxonomic sources (del Hoyo et al., 2014) split *Erythropitta erythrogaster* into multiple species, of which we sampled two]. We also sampled 81 specimens of 30 species (out of 35 currently recognized) of Asian barbets, 55 specimens of 12 species of *Psittacula* (one species was not sampled), and 57 specimens of 16 species of sandgrouse. Although our dataset did not include some species, we sampled the majority of recognized species in each family. Missing species are mostly qualitatively similar in plumage to the other species sampled, and we consider their addition unlikely to alter the broad patterns we observe (Supplementary Data). Where possible, we measured specimens collected relatively recently (Armenta et al., 2008), male and female specimens of sexually dichromatic species, and distinct subspecies (also see Supplementary Data) to obtain a comprehensive estimate of the color space occupied by each clade. These clades were selected partly because they are all monophyletic, but also because they are ecologically diverse, representing environments from forest understory all the way to arid environments. This phylogenetic and ecological disparity allows us to potentially make broader inferences about the evolution of bird color.

A particular concern with such studies is fading of specimens, which may alter plumage spectra. To offset this, we followed other studies in (1) selecting specimens with no qualitative evidence of fading and (2) where possible, measuring specimens of diverse collection ages (Armenta et al., 2008; Doutrelant et al., 2016; Stoddard and Prum, 2008), including relatively recent specimens (recent implying the 1960s onwards until 2014). The tetrahedral color space does not perform well on black patches (and may artificially inflate the relative photon catch of color channels, depending on specimen condition), and the TetraColorSpace program therefore treats these patches as possessing zero reflectance.

### Reflectance spectrometry and photon catch of color cones

We measured plumage reflectance of museum skins, using an S2000 UV-visible fiber-optic reflectance spectrophotometer (Ocean Optics, Inc., Dunedin, FL, USA) with a DT1000 deuterium-tungsten halogen light source. Measurements were referenced to a CIE D65 (white under average daylight illumination) white standard (Milton Roy Color Products, Rochester, NY, USA), and dark referenced to a black surface. We first moved the probe over each region of the body, looking at the computer display to ensure that (to the best of our ability) we did not miss any patches that are not visible to the human eye (particularly cryptic UV sexual dimorphism). We then measured one reflectance spectrum for each color patch on each specimen using the Overture (Ocean Optics, Inc.) software. Although our dataset did not take within-patch variation into account as a result, we generally observed that intraspecific variation (and qualitatively, within-patch variation observed by moving around the probe) for the same patch was lower than interspecific variation, and is thus unlikely to alter the patterns we observe. Using photographs of each specimen (taken in lateral, dorsal and ventral perspectives), we divided the body of the bird into seven regions commonly used by ornithologists to describe plumage patterns: the crown (which we defined as all patches occupying the upper surface of the head to above the eye, extending backward to the nape), the cheek (defined as all patches occupying the region from the eye downwards to the base of the mandible, extending backward to the base of the neck), the throat (all patches below the base of the mandible), the upperparts (the back and rump), wing, tail (typically the upper surface, but see below), and underparts (breast, belly and vent) (Dale et al., 2015). For *Psittacula* parakeets, we did not treat the throat as a separate region from the cheek, owing to the structure of plumage patterns across their heads (briefly, the throat is a restricted region of overall head area in this genus, continuous with the black cheek stripes; these were included instead in our analyses of cheek patterns as they extend up the sides of the head). Secondly, owing to the long tails of these parakeets, we could obtain reliable measurements of undertail colors in all species, and these are therefore included in our analyses. For reasons of accessibility due to the methods of skin preparation, we did not measure the underwing and undertail colors of any other species except parakeets, and we could not reliably access the white wing patches or tails of certain Pittidae, which have also therefore been excluded from our analyses (these, however, are qualitatively similar across species, and likely serve a conspicuousness function during displays, but are not often visible in the perched bird) (Erritzoe and Erritzoe, 1998). Finally, our analyses considered only plumage colors and not the colors of bare parts such as legs, bills or facial skin, owing to concerns about fading, specimen preservation and therefore the measurement of luminance. We do, however, include the bills of the *Psittacula* parakeets (largely bright red, with the exception of two species with orange bills, and black in the females of some species) in our tetrahedral plots of color space, but not in subsequent analyses (this is because specimen preparation may alter the brightness of color in some specimens).

We used the MATLAB (MathWorks, Inc., Natick, MA, USA) program TetraColorSpace (Stoddard and Prum, 2008) and the R (R Core Team, 2013) package PAVO (Maia et al., 2013) to analyze reflectance spectra. These algorithms incorporate cone sensitivities for averaged VS and UVS avian visual systems, to calculate the theoretical photon catch for each cone (this representing the signaler phenotype, or visual signal under idealized light conditions (Stoddard and Prum, 2008). Although the use of averaged visual systems does not directly model perception for each species, photon catch provides an objective way to quantify spectral signal in different portions of the avian-visible spectrum (Burkhardt, 1989; Endler and Mielke, 2005; Goldsmith, 1990). We calculated photon catch of the four color cones (VS models for pittas and barbets, UVS models for parakeets and sandgrouse) using both programs (the values were concordant across both), performing the von Kries correction (Vorobyev and Osorio, 1998) using a uniform white light (or idealized light) spectrum. Birds process luminance information separately from color information (Endler and Mielke, 2005; Vorobyev and Osorio, 1998), using the double cones (Goldsmith and Butler, 2005). Thus, we also used PAVO to calculate the photon catch of the double cones as a measure of luminance, using known sensitivities for the double cone of the blue tit (*Cyanistes caeruleus*) (Hart et al., 2000). Again, although this does not directly represent luminance perception by each species, it provides an objective comparison of luminance differences in plumage. Using the relative photon catch values for each cone, we visualized plumage colors of each bird family in Goldsmith's tetrahedral color space (Burkhardt, 1989; Goldsmith, 1990). We also calculated the percentage volume of total avian color space occupied, using published measures of the latter (Stoddard and Prum, 2011) as a reference.

### Analyses

After obtaining raw photon catch values for each cone, we transformed these values into a three-dimensional XYZ color space representing the receptor-noise limited model of tetrachromatic color vision (Siddiqi et al., 2004; Vorobyev, 2003; Vorobyev and Osorio, 1998; Vorobyev et al., 1998). This was accomplished using the Weber fraction of each cone, which is calculated using the signal:noise ratio and the relative abundance of each cone in the retina. We incorporated published Weber fractions of the four cones for *Leiothrix lutea* (Vorobyev et al., 1998) as described in the literature (Cassey et al., 2008; Delhey et al., 2015), to transform photon catch values for each color patch into XYZ coordinates using MATLAB. The advantage of this color space is that distances between points are expressed in just noticeable differences (JND), an indication of the perceptual distance between them (Cassey et al., 2008; Pike, 2012; Siddiqi et al., 2004; Vorobyev and Osorio, 1998), thus providing a better approximation of how differences in color are perceived by the avian visual system. We also plotted color distributions for each family in this color space using the RGL package (Adler et al., 2003) in R *sensu* (Delhey et al., 2015).

First, we estimated the proportion of variation in coordinate space explained by the first major axis using principal components analysis (PCA) on the XYZ coordinates obtained above, following published studies (Cassey et al., 2008; Delhey et al., 2015). We used PCA only to estimate the proportion of variance along this line, and not in any subsequent analysis. In order to quantify the presence of complementary colors, we required a metric that included not only the distance of each color from the origin, but which distinguished colors lying on opposite sides of the achromatic point (information which is lost in Euclidean distance measures). To achieve this, we transformed the XYZ coordinates into a three-dimensional spherical coordinate space in MATLAB, with the achromatic point at the origin. We used the elevational coordinate Φ (in radians) from this spherical coordinate space as a ‘color score’ in subsequent analyses (using a species average, also see Results). This emerged from our analyses (see Results), and enabled us to look at the linear distribution of scores with respect to the achromatic point. Therefore, colors on opposite sides of the achromatic point should exhibit scores with opposite signs (Endler, 1992; Endler and Mielke, 2005), and thus exhibit little spectral overlap (Ham and Osorio, 2007). Additionally, this enabled us to transform complex measurements of color space into a ‘trait’ that could be compared using comparative phylogenetic analyses. First, we constructed histograms of the maximum and minimum color score for each distinct taxon (species and distinctive subspecies) within a family, and compared them using *t*-tests (see Results for sample sizes). First, we performed one-sample *t*-tests on the maximum and minimum values to test whether they differed from a mean of zero, and then a paired two-sample *t*-test to test whether they differed from each other. These two tests together served to test whether each clade possessed colors lying on opposite sides of the achromatic point (zero).

Finally, we used phylogenetic comparative analyses to investigate whether color and luminance scores across each body region exhibited phylogenetic signal. We first sorted all the patches measured in each of the four avian clades into crown, cheek, throat, back, wing, tail and underpart patches (except the parakeets, where we measured crown, cheek, back, wings, underparts and both upper and undertail) for both males and females. Next, we calculated the average color score and luminance index (double cone photon catch) for each region of the body for the male plumage of each species (to account for some species possessing more color patches than others, and thus enable direct comparisons). Using published phylogenetic information for each family (Den Tex and Leonard, 2013; Groombridge et al., 2004; Irestedt et al., 2006; Jetz et al., 2012; Kundu et al., 2012) and the ape and phytools (phylosig function) packages (Paradis et al., 2004; Revell, 2012) in R, we calculated Pagel's λ, a measure of phylogenetic signal, for color and luminance scores of each body region. This index measures whether trait evolution (in this case, color and luminance scores) follows a Brownian motion model of evolution, where phylogenetic effects drive trait evolution. In this scenario, the λ value is 1, whereas departures from Brownian motion result in a value lower than 1 (Münkemüller et al., 2012; Pagel, 1999). To estimate the significance of the measured statistic, we compared this value to 1000 randomized values obtained using the inbuilt functions of the phytools package. To further verify these results, we additionally performed a second analysis. Using a phylogenetic distance matrix derived from the ape package, we calculated Mantel correlations between this matrix and an interspecific trait distance matrix derived for color and luminance for each body region (see Supplementary Data). This essentially calculated the pairwise difference in average color score between each pair of species, for each body region. Thus, we obtained a matrix of ‘color distances’ that could be correlated to the phylogenetic distance matrix. This test provided additional quantification on the effects of phylogenetic relatedness on interspecific color variation, and we predicted that a lack of signal, together with the presence of complementary colors, would be consistent with pattern diversification by redistributing a complementary color pair across body regions.

## Supplementary Material

Supplementary information
