# Supplementary material for: Visual signal evolution along complementary color axes in four bird lineages
Source: Biol Open. 2020 Sep 18;9(9):bio052316. doi: 10.1242/bio.052316 (PMC7520455; doi:10.1242/bio.052316)
Supplement: Supplementary information [file biolopen-9-052316-s1.pdf]

Biology Open (2020): doi:10.1242/bio.052316: Supplementary information

Color Space Occupancy

Erythropitta and *Hydrornis* males combined

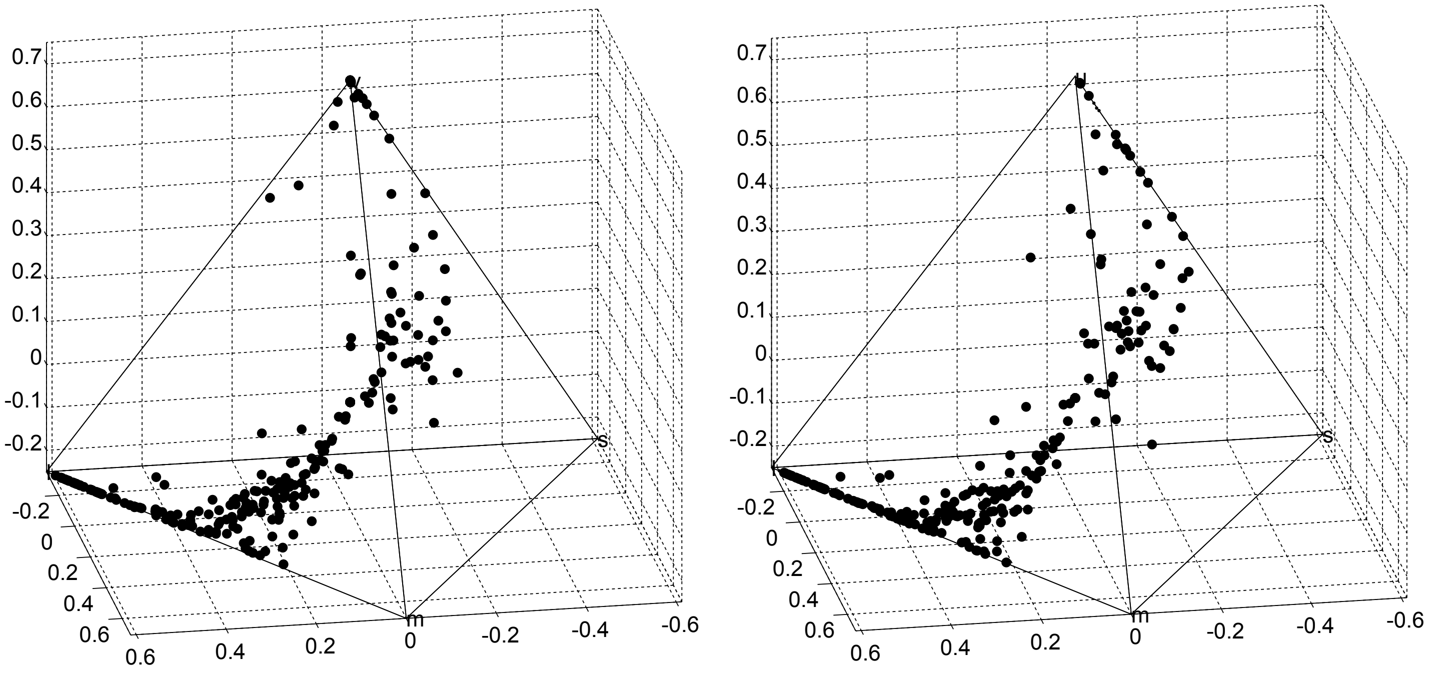

VS UVS

Figure S1: Comparison of color space occupancy in pittas modeled using VS and UVS visual systems

Biology Open • Supplementary information

Biology Open (2020): doi:10.1242/bio.052316: Supplementary information

Supplementary Data

Click here to Download Supplementary Data

Biology Open • Supplementary information
